# Passive Blood-Flow-Restriction Exercise’s Impact on Muscle Atrophy Post-Total Knee Replacement: A Randomized Trial

**DOI:** 10.3390/jcm14155218

**Published:** 2025-07-23

**Authors:** Alexander Franz, Luisa Heiß, Marie Schlotmann, Sanghyeon Ji, Andreas Christian Strauss, Thomas Randau, Frank Sebastian Fröschen

**Affiliations:** 1 Department of Orthopedics and Trauma Surgery, University Hospital Bonn, 53127 Bonn, Germany; s4luheis@uni-bonn.de (L.H.); s4meschl@uni-bonn.de (M.S.); andreas.strauss@ukbonn.de (A.C.S.); frank.froeschen@ukbonn.de (F.S.F.); 2 Department of Trauma and Orthopedic Surgery, BG Klinik Ludwigshafen, 67071 Ludwigshafen, Germany; 3 Section Exercise Physiology, Institute of Exercise Training and Sport Informatics, German Sport University Cologne, 50933 Cologne, Germany; s.ji@dshs-koeln.de; 4 German Research Centre of Elite Sport, German Sport University Cologne, 50933 Cologne, Germany; 5 Department of Orthopedics, Orthopedic Surgery and Sports Medicine, Augustinian Hospital Cologne, 50678 Cologne, Germany; trandau@severinskloesterchen.de

**Keywords:** knee arthroplasty, occlusion training, rehabilitation, physical function

## Abstract

**Background/Objectives**: Total knee arthroplasty (TKA) is commonly associated with postoperative muscle atrophy and weakness, while traditional rehabilitation is often limited by pain and patient compliance. Passive blood flow restriction (pBFR) training may offer a safe, low-threshold method to attenuate muscle loss in this early phase. This pilot study examined the feasibility, safety, and early effects of pBFR initiated during hospitalization on muscle mass, swelling, and functional recovery after TKA. **Methods**: In a prospective, single-blinded trial, 26 patients undergoing primary or aseptic revision TKA were randomized to either a control group (CON: sham BFR at 20 mmHg) or intervention group (INT: pBFR at 80% limb occlusion pressure). Both groups received 50 min daily in-hospital rehabilitation sessions for five consecutive days. Outcomes, including lean muscle mass (DXA), thigh/knee circumference, 6 min walk test (6 MWT), handgrip strength, and patient-reported outcomes, were assessed preoperatively and at discharge, six weeks, and three months postoperatively. Linear mixed models with Bonferroni correction were applied. **Results**: The INT group showed significant preservation of thigh circumference (*p* = 0.002), reduced knee swelling (*p* < 0.001), and maintenance of lean muscle mass (*p* < 0.01), compared with CON, which exhibited significant declines. Functional performance improved faster in INT (e.g., 6 MWT increase at T3: +23.7%, *p* < 0.001; CON: −7.2%, n.s.). Quality of life improved in both groups, with greater gains in INT (*p* < 0.05). No adverse events were reported. **Conclusions**: Initiating pBFR training on the first postoperative day is feasible, safe, and effective in preserving muscle mass and reducing swelling after TKA. These findings extend prior BFR research by demonstrating its applicability in older, surgical populations. Further research is warranted to evaluate its integration with standard rehabilitation programs and long-term functional benefits.

## 1. Introduction

Total knee arthroplasty (TKA) is one of the most frequently performed surgeries worldwide, with a significant impact on joint mobility and subjective pain [1]. However, postoperative recovery after TKA is often associated with persistent muscular atrophy and weaknesses [2,3]. This is particularly pronounced in patients receiving a revision of a TKA due to infection (periprosthetic joint infection, PJI) or aseptic loosening, since the second operative intervention causes even higher immobility in the postoperative phase [4]. An attempt to counteract this complication by early physiotherapy often fails due to increasing postoperative pain [5], highlighting the need for interventions that provide sufficient muscular stimulus while ensuring patient compliance.

The standard postoperative phase after TKA in Germany is characterized by a hospitalization of at least three to five days with daily physiotherapy, three weeks of in- or outpatient rehabilitation followed by long-term supervised physiotherapeutic therapy (up to one year) [6]. From a global perspective, this rehabilitation approach differs significantly from those in other industrialized countries (e.g., the USA and Canada) without showing better functional results after one year [7]. In this context, long-term follow-ups show that persistent muscle atrophy and reductions of muscle strength are the predominant reasons for reduced functionality and satisfaction after TKA [8].

Although current surgical approaches try to prevent direct muscle damage, molecular analysis from muscle biopsies has revealed an indirect impact on muscle physiology by showing that protein synthesis is downregulated and concurrently the expression of key atrophy genes is upregulated. An analysis of vastus lateralis muscle samples during TKA showed a blocked protein synthesis capacity by reduced building of the translation initiation complexes with concurrent upregulation of FoxO3a products (e.g., Muscle RING-finger protein-1, MURF-1), enhancing muscular protein breakdown during and after surgery [9,10]. In light of these findings, early postoperative interventions should aim to counteract these catabolic processes effectively and, ideally, should be broadly applicable for every patient (independent of the activity level after surgery) and resource-efficient in clinical practice.

Blood flow restriction training (BFR) is an exercise technique increasingly relevant for clinical practitioners, physicians, and surgeons. By using specific tourniquets around the proximal parts of the upper or lower extremities, BFR training is able to improve muscle mass and strength to a similar extent to high-load mechanical resistance training, while only low mechanical loads (20–30% of the one-repetition maximum) are applied [11]. BFR has already been applied successfully as a conservative treatment option in osteoarthritis patients [12], as a prehabilitative approach in TKA [13], or as an additional rehabilitation tool after orthopedic surgery [14].

In addition to the well-documented benefits of active BFR training, emerging evidence suggests that passive BFR (pBFR), in absence of any muscle contraction, may also attenuate muscle atrophy during periods of immobility [15] and in post-surgical clinical settings [16]. The proposed mechanism underlying this effect is an increase in intracapillary hydrostatic pressure, which may induce muscle cell swelling and trigger anabolic or even suppress catabolic signaling pathways [17]. This suggests that passive BFR could mitigate atrophy even in the absence of voluntary contraction, particularly in clinical populations with a heightened vulnerability to muscle loss. However, recent findings in young and healthy individuals have shown that pBFR did not favorably impact myofibrillar protein synthesis [18], although it may reduce muscle protein degradation (e.g., suppression of MURF1) [19], indicating that pBFR may offer a novel therapeutic strategy by potentially suppressing catabolic pathways in the early recovery phase after TKA. Another advantage of applying pBFR during hospitalization is its suitability for all patients, regardless of their activity or fitness levels, while also requiring fewer staff for implementation in comparison with active interventions. Moreover, unlike passive electrical stimulation [20], pBFR may not be adversely affected by postoperative edema formation.

To date, most clinical data on pBFR have been derived from relatively young and healthy populations (mainly after anterior cruciate ligament reconstruction). This raises the question of whether pBFR training is also suitable after major joint surgeries (e.g., primary or revision TKA) and older people with comorbidities and limited mobility, as is the case for a majority of patients undergoing TKA. Therefore, the present study tests the hypothesis that a pBFR intervention initiated immediately after TKA is feasible and safe, and that it supports the preservation of muscle mass by improving functional recovery during the early postoperative phase. The specific objectives of this study are (1) to evaluate the feasibility and safety of pBFR when started on the first postoperative day, and (2) to assess its impact on muscle mass preservation, joint swelling, and early functional recovery.

## 2. Materials and Methods

### 2.1. Patients and Methods

Twenty-six patients (ten male, sixteen female; age: 63.8 ± 10.5 y; height: 168.3 ± 10.3 cm; weight: 92.2 ± 15.9 kg; BMI: 32.68 ± 6.1) with either primary knee osteoarthritis undergoing TKA (*n* = 21) or aseptic loosening of a TKA undergoing revision TKA (rTKA, *n* = 5) participated. Inclusion criteria were as follows: indication for primary TKA due to end-stage gonarthrosis; indication for revision TKA due to aseptic loosening of the existing implant, with no evidence of bacterial growth detected in preoperatively performed knee joint aspirations; ability to perform the exercises of the measurement battery; and approval for surgery by the anesthetic department and written informed consent for participation in the study. Exclusion criteria were as follows: absence of iatrogenic changes in the vascular system of the lower limbs (e.g., stenting, bypassing); sickle cell anemia; and infections or open wounds of the lower extremities. Participants were randomly assigned to one of two groups:

1. Control group (CON, Primary TKA: 11, Revision TKA: 2):

Standard postoperative care + sham BFR (20 mmHg) once daily.

2. Intervention group (INT, Primary TKA: 10, Revision TKA: 3):

Standard postoperative care + passive BFR (80% limb occlusion pressure, LOP) once daily.

Randomization was performed using a computer-generated list (www.randomizer.org, accessed on 4 July 2022). Allocation was concealed using sealed, opaque, sequentially numbered envelopes, prepared by a researcher not involved in recruitment or data collection. All patients provided informed consent prior to participation. Envelopes were opened after participants gave informed consent. The study was approved by the local Ethics Committee (Trial-ID: 043/22) and conducted in accordance with the Declaration of Helsinki.

### 2.2. Study Design

The study design consists of a prospective, single-blinded, parallel study design to determine the influence of a pBFR-intervention as an immediately postoperative treatment strategy during hospitalization. While both groups underwent routine postoperative treatment in the hospital, an additional pBFR-intervention was applied bilaterally on the lower limbs once per day, beginning at the first postoperative day with differences in the applied pressure between the two groups.

### 2.3. Sample Size Calculation

Due to the lack of directly comparable reference data, a priori effect size estimation was not feasible. Therefore, post hoc power analyses were conducted based on the observed effect sizes and variance components derived from the fitted linear mixed-effects models (simr package, R Core team (Quelle), Version 4.4.2). The results indicated that the available sample size (*n* = 13 per group) provided sufficient statistical power to detect group-by-time interaction effects for muscular outcomes—such as thigh circumference, lean mass of the operated and nonoperated leg, and knee swelling—with a mean power of 87.5% (range: 74.5–100%). In contrast, functional outcomes such as the 6 MWT and CRT showed insufficient power (range: 21.6–55.4%).

### 2.4. Standard Operative and Postoperative Treatment

#### 2.4.1. Surgery

All patients with primary osteoarthritis underwent posterior cruciate retaining bicondylar knee arthroplasty with TiN-coated fixed-bearing cruciate retaining protheses (advanced coated system (ACS) FB system^®^; Implantcast GmbH, Buxtehude, Germany). Patients with aseptic loosening and the need for revision surgery underwent one-stage exchange with the implantation of a TiN-coated hinged TKA (MUTARS GenuX MK Revision Knee System^®^, Implantcast GmbH, Buxtehude, Germany). In all patients a “standard approach” with longitudinal midline incision and medial parapatellar arthrotomy was performed. All surgeries were performed by certified senior arthroplasty surgeons. During hospitalization, all included patients received standardized anticoagulation prophylaxis by 4000 I.E. of enoxaparin sodium per day (Clexane 4000 I.E. 40 mg/0.4 mL).

#### 2.4.2. Daily Physical Therapy

Daily physiotherapy during hospitalization included mobilization, gait training, and an active and passive range of motion exercises routinely associated with prolonged passive motion using a continuous passive motion (CPM) device. Knee flexion was gradually increased to at least 90° before discharge.

#### 2.4.3. Rehabilitation

After hospitalization, all patients underwent a three-week inpatient or outpatient rehabilitation program, including daily supervised resistance training, proprioceptive exercises, and endurance sessions tailored to individual recovery goals, consistent with German national guidelines for post-TKA rehabilitation [21].

### 2.5. Interventions

#### 2.5.1. Limb Occlusion Pressure and Blood Flow Restriction

**Figure 1:**
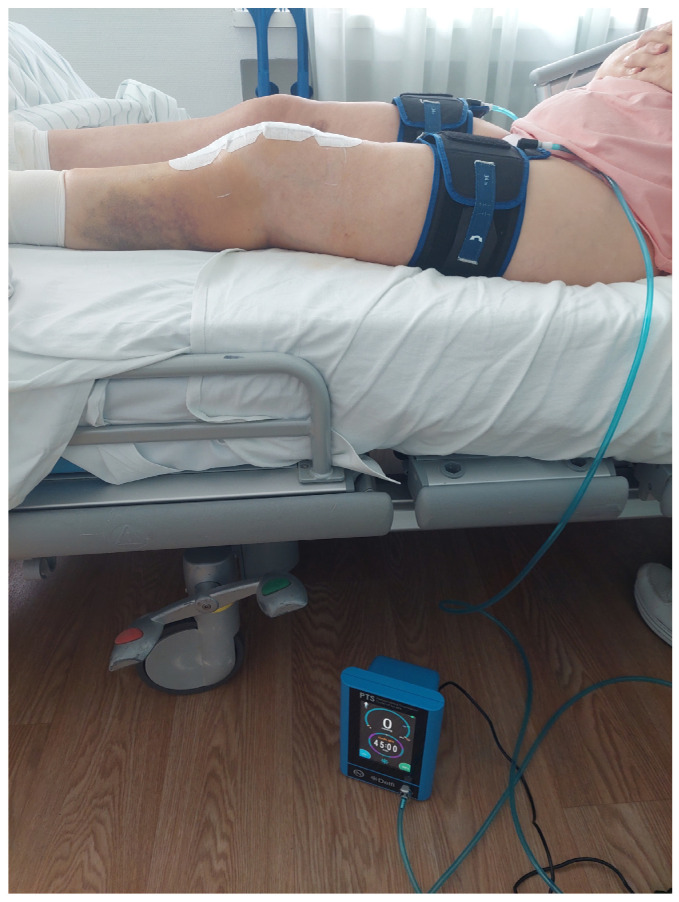
Example of a passive blood flow restriction application after total knee arthroplasty surgery during the hospitalization phase.

To determine the applied external pressure during pBFR training, the individual limb occlusion pressure (LOP) was measured daily using pneumatic tourniquets (11.5 cm width; Delfi Medical Innovations Inc., Vancouver, BC, Canada) placed proximally on each thigh before the training session. Within this regard, the inflatable tourniquet of 11.5 cm width was placed proximal on both thighs before the training session (PBFR, Delfi medical Inc., Vancouver, BC, Canada). Following a 10 min rest in a supine position, LOP was automatically determined (Figure 1).

#### 2.5.2. In-Hospital Rehabilitation Protocol

All enrolled patients received during their hospitalization an in-hospital rehabilitation protocol that consisted of a daily pBFR training session that lasted 50 min. For exercise, patients stayed in bed in a lying rest position and the inflatable tourniquets were placed proximal on each leg. The training protocol was performed on both legs alternating. On each site there were five occlusion intervals that lasted five minutes and after each of them a reperfusion interval that lasted five minutes as well. When the occlusion was started on the first leg, the other stayed deflated and was inflated when the reperfusion interval started on the first leg. The applied pressures for pBFR were for CON 20 mmHg and for INT 80% of the LOP (mean: right: 172.6 mmHg ± 30.6, left: 170.0 ± 30.4 mmHg) [22,23,24]).

### 2.6. Outcome Measures

The outcomes measures were obtained two days before surgery (T0), post-hospitalization (T1, mean 6.5 ± 0.9 days post-surgery), and six weeks (T2) as well as three months (T3) post-surgery. The examination battery consists of anthropometric data (body height, body weight), muscle mass and function analysis, overall functional tests, and questionnaire-based data.

Quadriceps and thigh muscle assessment: Thigh circumference was measured at mid-distance between the spina iliaca anterior superior and patella [13]. Lean body mass was measured using dual-energy X-ray absorptiometry (DXA, Horizon W DXA System, Hologic Medicor GmbH, Berlin, Germany), calibrated daily with a manufacturer-provided spine phantom. Regional analysis was used to assess the lean mass of the operated and nonoperated thigh based on standardized anatomical landmarks [25]. Femur mass was not separately analyzed. Knee swelling was recorded via mid-patella circumference. Hand grip strength (Baseline Hydraulic Hand Dynamometers, LiTE, New York, NY, USA)) was measured bilaterally.

Functional assessments: The six-minute walk test (6 MWT) is a submaximal exercise test that measures the total distance a patient can walk on a flat, hard surface in six minutes. It assesses functional endurance and aerobic capacity, particularly in populations with mobility limitations or recovering from surgery [26]. The chair rise test (CRT) recorded the number of sit-to-stand repetitions in 30 s [27].

Questionnaires: Pain and quality of life were evaluated using the Knee Injury and Osteoarthritis Outcome Score (KOOS) and short-form 36 score (SF-36), and a visual analog scale (VAS) for pain intensity.

All outcome assessors were blinded to group allocation. Intervention sessions were conducted separately from assessment areas, and participants were instructed not to share group information.

### 2.7. Statistics

For statistical analysis, R (R Core team (Quelle), Version 4.4.2) war used. Homoscedasticity and normality were visually assessed via residual and Q-Q plots. Linear mixed models (lme4 package) were used to analyze longitudinal changes (fixed effect, with 3 to 4 levels) and between groups (fixed effect, with 2 levels, i.e., CON vs. INT). To account for interindividual differences at baseline, a random intercept for each participant (i.e., the participant’s own baseline of each measure) was included. If significant main effects of time, group, or their interaction were detected, post hoc pairwise comparisons were conducted (emmeans package), with Bonferroni correction applied to adjust for multiple testing. These comparisons were used to determine which specific factor levels differed significantly from one another. For pairwise comparisons (within-group change and between-group difference), Cohen’s effect size (*d*) and 95% confidence intervals (95% CI) were determined by means of the effsize package. A *d* of ≥0.8 was referred to large, 0.5 ≤ *d* < 0.8 moderate, and 0.2 ≤ *d* < 0.5 small effect [28]. Significance was set at *p* < 0.05. Results are reported as mean ± standard deviation.

## 3. Results

### 3.1. Quadriceps and Thigh Muscle Assessment

**Figure 2:**
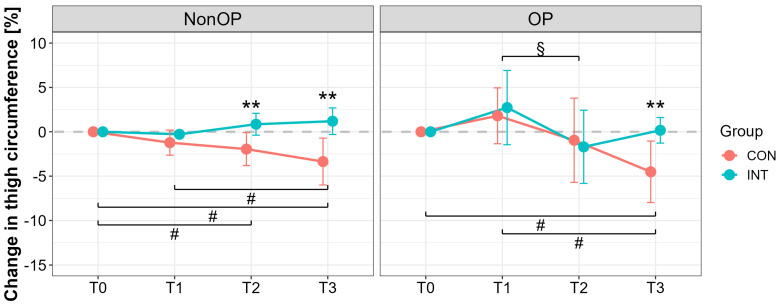
Percentage changes in thigh circumference for the operated (OP) and nonoperated leg (NonOP) during the pre- and postoperative periods. Data are provided as mean ± standard deviation. T0, before surgery; T1, post-hospitalization; T2, six weeks post-surgery; T3, three months post-surgery; CON, control group; INT, intervention group. ^#^ *p* < 0.01, significant difference between time points within CON ^§^ *p* < 0.01, significant difference between time points within INT, ** *p* < 0.01, significant difference between groups.

At T0, mean thigh circumference (OP leg) was similar between groups (CON: 65.0 ± 9.7 cm; INT: 65.8 ± 9.2 cm). Significant effects were found for time (*p* < 0.001) and time × group interaction (*p* = 0.011), but not for group (*p* = 0.173). Thigh circumference of the NonOP leg showed significant effects for time (*p* = 0.038), group (*p* < 0.001), and interaction (*p* < 0.001). At T3, significant reductions were observed only in CON (OP leg with a large effect: T3 vs. T0, *p* = 0.004, *d* = 1.84, 95% CI [0.60, 3.03]), with significant group differences (T3, *p* = 0.002, *d* = 1.76, 95% CI [0.70, 2.79]) (Figure 2).

**Figure 3:**
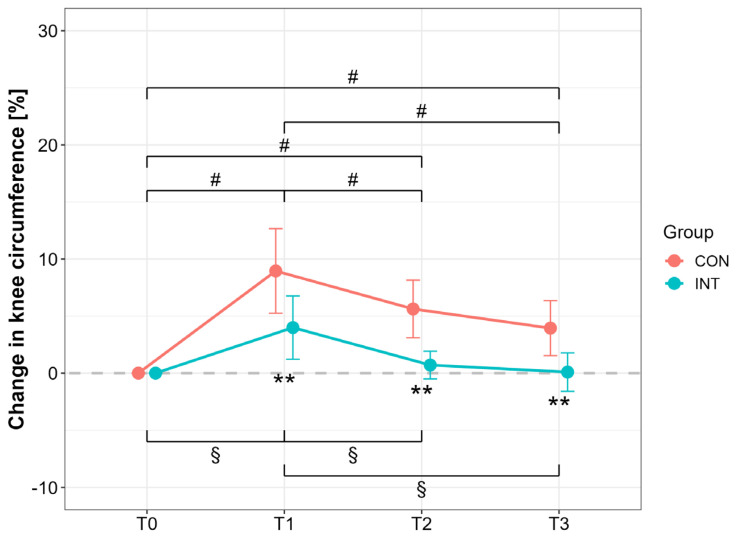
Percentage changes in knee circumference for the operated (OP) and nonoperated leg (NonOP) during the pre- and postoperative periods. Data are provided as mean ± standard deviation. T0, before surgery; T1, post-hospitalization; T2, six weeks post-surgery; T3, three months post-surgery; CON, control group; INT, intervention group. ^#^ *p* < 0.01, significant difference between time points within CON ^§^ *p* < 0.01, significant difference between time points within INT, ** *p* < 0.01, significant difference between groups.

Knee circumference increased postoperatively in both groups (T1 vs. T0, *p* < 0.001; CON: *d* = 3.42, 95% CI [1.62, 3.42]; INT: *d* = 2.04, 95% CI [0.74, 3.28]), but to a lesser extent in INT (*p* < 0.001; CON vs. INT: *d* = 1.52, 95% CI [0.49, 2.50]). At T3, only INT returned to baseline (T0 vs. T3, *p* = 1.00, *d* = −0.08, 95% CI [−0.95, 0.80]), while CON remained elevated (T0 vs. T3, *p* < 0.001, *d* = −2.32, 95% CI [−3.45, −1.14]). Between-group differences were significant at both T2 and T3 (*p* < 0.001), with large effects (T2: *d* = 2.48, 95% CI [1.17, 3.75]; T3: *d* = 1.85, 95% CI [0.75, 2.92]) (Figure 3).

**Figure 4:**
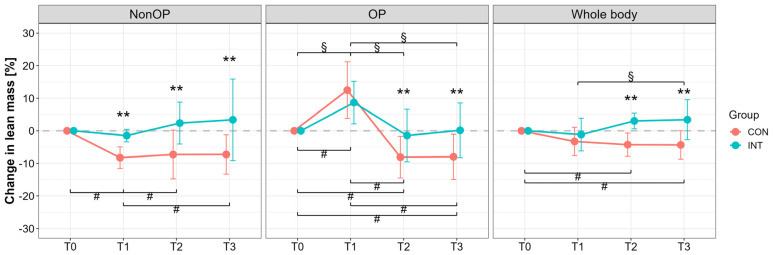
Percentage changes in lean mass assessed by dual-X-ray absorptiometry (DXA) measurement for the operated (OP), nonoperated leg (NonOP), and whole body during the pre- and postoperative periods. Data are provided as mean ± standard deviation. T0, before surgery; T1, post-hospitalization; T2, six weeks post-surgery; T3, three months post-surgery; CON, control group; INT, intervention group. ^#^ *p* < 0.01, significant difference between time points within CON ^§^ *p* < 0.01, significant difference between time points within INT, ** *p* < 0.01, significant difference between groups.

Lean mass (DXA) of the OP leg at T0 was 7961 ± 1844 g (CON) and 8636 ± 1287 g (INT); NonOP leg: 8445 ± 2162 g (CON), 8579 ± 1143 g (INT). For the NonOP leg, significant group (*p* < 0.001) and interaction effects (*p* = 0.02) were observed, with large reductions only in CON from T1 to T3 compared with T0 (*p* ≤ 0.05, T0 vs. T1: *d* = 3.54, 95% CI [1.69, 5.35]; T0 vs. T2: *d* = 1.38, 95% CI [0.27, 2.43]; T0 vs. T3: *d* = 1.70, 95% CI [0.50, 2.84]) (Figure 4). OP leg lean mass in INT remained stable (*p* = 1.00, T0 vs. T2: *d* = 0.25, 95% CI [−0.64, 1.13]; T0 vs. T3: *d* = −0.03, 95% CI [−0.90, 0.85]), while CON showed significant declines with large effects (*p* < 0.01, T0 vs. T2: *d* = 1.79, 95% CI [0.57, 2.96]; T0 vs. T3: *d* = 1.62, 95% CI [0.45, 2.75]). Whole-body lean mass declined in CON *p* ≤ 0.05, T0 vs. T2: *d* = 1.69, 95% CI [0.50, 2.82]; T0 vs. T3: *d* = 1.41, 95% CI [0.30, 2.47]), but increased in INT with a large effect (T1 vs. T3, *p* = 0.03, *d* = −0.81, 95% CI [−1.72, −0.11]). Group differences were significant at T2 and T3 (*p* < 0.001), with large effects (T2: *d* = 2.37, 95% CI [1.20, 3.54]; T3: 1.46, 95% CI [0.45, 2.44]).

### 3.2. Functional Assessments

Hand grip strength decreased post-surgery in both groups with large effects (T1 vs. T0: CON: −11.6 ± 6.6%, *d* = −2.48, 95% CI [−3.18, −1.78]; INT: −9.7 ± 3.8%, *d* = −3.59, 95% CI [−4.49, −2.68]; *p* < 0.001 for both groups), but recovered to baseline only in INT by T3 (1.5 ± 7.9%, *d* = 0.27, 95% CI [0.18, 0.71]; *p* = 1.00), while CON remained reduced (−4.2 ± 10.9%, *d* = −0.54, 95% CI [−0.99, −0.08]; *p* = 0.003; group difference at T3: *d* = 0.59, 95% CI [0.14, 1.04], *p* = 0.05).

The 6 MWT distance at T0 was 452 ± 195 m (CON) and 431 ± 167 m (INT). At T1, performance dropped significantly in both (CON: −68.5 ± 20.3%, *d* = −4.77, 95% CI [−5.91, −3.62]; INT: −60.2 ± 19.0%, *d* = −4.48, 95% CI [−5.56, −3.39]; *p* < 0.001 for both groups). At T3, INT improved above baseline (23.7 ± 46.1%, *d* = 0.73, 95% CI [0.26, 1.19], *p* < 0.001), while CON did not (−7.2 ± 23.3%, *d* = −0.44, 95% CI [−0.88, 0.01], *p* = 0.557) (group difference at T3: *d* = 0.85, 95% CI [0.39, 1.30], *p* < 0.001).

CRT performance improved in both groups (T3 vs. T0: CON: +21.3 ± 55.2%, *d* = 0.55, 95% CI [0.03, 1.06]; INT: +20.0 ± 29.3%, *d* = 0.97, 95% CI [0.43, 1.50]; *p* < 0.001), with no significant group or interaction effects (*p* ≥ 0.919).

### 3.3. Questionnaires

As summarized in Table 1, all KOOS measures showed a significant main effect of time; however, no significant main effect of group or time × group interaction was found. Further analysis revealed significant improvements in all sub-parameters of KOOS compared with baseline (i.e., T0) at T2 (*p* < 0.05, except for SportRec, *p* = 1.00) and T3 (*p* < 0.05).

In SF-36, most subdomains showed time effects (*p* ≤ 0.045), especially in the physical health dimension (PCS) and mental health dimension (MCS) at T2. Group differences were only detected for bodily pain (BP, *p* < 0.05) (Table 2). No significant adverse events occurred, and all participants completed the full intervention protocol.

There were no adverse events during the study (e.g., no postoperative hematomas, wound-healing disorders or revisions of any etiology). Furthermore, all included subjects were able to complete the full intervention protocol (no dropouts).

## 4. Discussion

The present pilot trial is the first one to analyze the effects of a pBFR intervention on muscle mass, postoperative swelling, and functionality after elective TKA surgery. The results show that the application of a pBFR intervention starting at the first day after TKA surgery is a safe and patient-compliant intervention strategy after primary and revision TKA, preserving muscle mass on the operated and nonoperated leg by simultaneously reducing postoperative swelling of the knee joint. However, the muscle preservation effect of the INT was not associated with superior results in functional outcomes assessed by the 6 MWT, CRT, and QoL.

### 4.1. Muscle Atrophy After Total Knee Arthroplasty

Postoperative muscle atrophy is a usual side effect after TKA surgery, leading to significant muscle mass reductions of up to 14% of the operated leg and up to 7% in the nonoperated leg during the first seven days after surgery [29]. These reductions are associated with a decline in muscle strength [30] and neuromuscular activation [31] and are evident up to several years after surgery [32]. These postoperative declines are even more pronounced after revision TKA [33,34], basically due to the higher morbidity of the patients [35], the difficulty of the surgical intervention [36], and higher complication rates [37]. Since traditional rehabilitation protocols fail to counteract these pathological conditions [7], early postoperative interventions are necessary to reduce catabolic muscle conditions caused by the surgery itself [9,10].

To date, no prior studies have investigated the effects of pBFR immediately following TKA. The present pilot trial shows for the first time that the application of pBFR cycles, starting at the first day after TKA surgery, is able to maintain preoperative muscle mass condition after 6 weeks (muscle atrophy at operated leg: CON: −8 ± 6%; INT: −1 ± 8%; reduction of ~7%) and 3 months postoperative in comparison with a sham intervention, which shows the typical postoperative muscle atrophy (Figure 2 and Figure 4). While acute swelling in the operated thigh may temporarily mask early protective effects (Figure 4), the preservation of lean mass in the nonoperated limb suggests systemic or crossover effects likely mediated by early protective mechanisms (muscle mass of the nonoperated leg at post-3 months: CON: −7 ± 6%; INT +3 ± 13%, reduction of ~11%) (Figure 2).

While the fundamental mechanisms of muscle atrophy are well described, the specific modulatory effects of pBFR in the postoperative setting remain insufficiently understood. However, muscle swelling, by BFR-induced rises in effective filtration pressure and corresponding fluid shifts, changes in metabolic homeostasis, as well as protein degradation, could be hypothesized [17,19,38]. Loenneke et al. (2012) [17] provided experimental support for the fluid shift hypothesis by reporting increased muscle thickness (rectus femoris and vastus lateralis) and associated reduced plasma volume by pBFR application in healthy subjects. These transient hemodynamic changes may contribute to anabolic signaling and muscle preservation.

Interestingly, two recent studies with healthy individuals failed to show a protective effect on muscle mass and strength in immobilization from bed rest [39] or from a knee brace [40]. However, these results stand in contrast to findings from clinical populations. For example, Takarada et al. reported an 11.3% attenuation of quadriceps muscle atrophy via pBFR in patients after ACL reconstruction [16], while Barbalho and colleagues showed a 6.2% reduction in knee extensor atrophy in critically ill patients undergoing pBFR during intensive care [24]. Although the present findings are in line with these previous reports in clinical populations, it can be assumed that pBFR may exert more pronounced effects in clinical populations characterized by high atrophy risk, advanced age, and reduced physical activity levels. This discrepancy in scientific findings across significantly different study cohorts (healthy individuals vs. surgical/patient populations) highlights the fact that results obtained from healthy subjects cannot be directly extrapolated to clinical populations. Within this regard, it seems that older patients especially are able to take advantage of BFR applications with considerably lower intensities (e.g., in prehabilitation [13]) or even without additional contraction protocols.

However, Iversen et al. [41] was unable to replicate these findings in physically active individuals following anterior cruciate ligament reconstruction. Notably, their study employed a non-pneumatic, non-regulating device and applied considerably lower pressures than used in the present study. This highlights the critical importance of methodological considerations in the application of BFR, particularly in interventions targeting clinical populations [42]. Equally, the underlying mechanisms and extent of muscle atrophy (such as the inhibition of protein synthesis and induction of atrophy-related signaling pathways) must be considered in such comparisons as well. It can be assumed that the physiological impact on skeletal muscle is more pronounced following major joint surgery than after arthroscopic procedures or simple immobilization.

### 4.2. Impact of Passive Blood Flow Restriction Training on Muscle Swelling

Similar to prolonged functional impairments, a greater tendency toward postoperative swelling has been associated with higher rates of patient dissatisfaction following TKA [43]. Furthermore, increased knee joint effusion has been identified as a contributing factor to chronic postoperative pain [44], with a reported prevalence of up to 35% among patients experiencing postoperative swelling [45]. These findings underscore the need for novel intervention strategies to complement existing multimodal approaches to pain and swelling management, ultimately aiming to improve postoperative outcomes and patient satisfaction after TKA.

The present results suggest that the early postoperative application of pBFR cycles is able to reduce joint swelling after 6 weeks and 3 months in comparison with our control condition (approximately 3–5% in all measured time points, Figure 3). These findings are well in line with the literature on postoperative treatment in radius fractures [46] and after ACL reconstruction [14]. The underlying mechanisms of this BFR-induced effect are largely unknown. Several physiological adaptations are speculated as a cause for this reductive swelling effect and are under investigation, such as supporting effects on tissue regeneration [38], increased debris removal by enhanced tissue perfusion [47], or beneficial impact on the inflammation cascade (as seen in reactive arthritis patients) [48].

### 4.3. Impact of Passive Blood Flow Restriction on Functional Outcomes and Quality of Life

While muscle mass, strength, and swelling are key clinical outcomes, recovery of physical function and quality of life (QoL) are of primary importance to patients. This study assessed functionality and subjective recovery via the 6 MWT, CRT, and QoL questionnaires (SF-36, KOOS). Previous research has shown that functional impairments persist for months after TKA despite standardized rehabilitation protocols [49]. In Germany, an extensive and resource-intensive rehabilitation program is standard; however, functional outcomes are not superior to those seen in countries with shorter programs (e.g., the USA, Canada) [7]. This suggests that current postoperative interventions may be delayed or insufficient to counteract surgical-induced impairments.

In the present pilot trial, the INT showed better preservation of muscle mass and reduced swelling, but no significant advantages in 6 MWT, CRT, or QoL outcomes in comparison to the sham intervention (Table 1 and Table 2). These findings align with previous work by Mizner et al. [50], indicating that preoperative physical condition plays a larger role in recovery than postoperative intervention. Supporting this, our previous research demonstrated that preoperative BFR training significantly enhanced postoperative muscle mass, strength, and QoL [13]. These results suggest that preoperative improvements in patients’ physical resources have a greater impact on recovery of function and QoL than early or late applied postoperative interventions.

However, by comparing mean changes with established minimal clinically important differences (MCIDs) for the 6 MWT (~26 m) [51], CRT Test (~2 repetitions) [52], KOOS-Pain- (≥12 points), and SF-36 bodily pain score (≥7 points for) [53], the INT group exceeded all thresholds (Δ6 MWT + 102 m; ΔChair-Stand + 3.1 reps; ΔKOOS-Pain + 16 pts; ΔSF-36 BP + 9 pts), whereas the CON group met only the chair-stand MCID. Thus, the muscle-preserving and anti-swelling effects of early pBFR are mirrored by functional gains that are clinically important and likely to reduce subsequent rehabilitation demands.

## 5. Summary and Clinical Impact

The present pilot trial shows for the first time that the application of a pBFR intervention, starting at the first day post-TKA surgery, is able to reduce muscle atrophy and joint swelling in comparison with a sham condition. Clinicians may consider implementing pBFR during the early postoperative period to support muscle preservation and manage swelling. It offers a practical, low-threshold adjunct to physiotherapy when conventional active rehabilitation is not yet feasible. Since the pBFR intervention can be reported as a safe, patient-compliant, and staff-friendly technique after primary and revision TKA surgery, its addition to daily physiotherapy or in combination with electrical stimulation could improve early rehabilitation after TKA. Based on the study findings, we propose a clinical implementation pathway involving daily in-bed pBFR cycles starting on postoperative day 1, with five-minute occlusion intervals (5 × 5 min per leg) at 80% LOP. The intervention should be applied once daily during hospitalization, with careful patient monitoring and staff training to ensure safety and compliance.

While our study uniquely investigates pBFR, previous clinical BFR applications typically involved low-load active exercise, showing significant improvements in muscle strength and function after TKA with minimal joint loading [54,55]. An alternative passive modality, neuromuscular electrical stimulation (NMES), has also demonstrated efficacy in reducing quadriceps atrophy and improving functional outcomes when applied early after TKA [56]. Therefore, future studies should try to evaluate the effects of pBFR in a broader patient population by simultaneously investigating the potential underlying mechanisms, potential side effects, and its applicability/comparability with additive, equivalent, or complementary treatment strategies (e.g., NMES). In summary, future investigations should include (1) large-scale RCTs with stratification for revision etiology (e.g., PJI vs. aseptic loosening); (2) studies assessing the molecular response (e.g., ubiquitin–proteasome activity) to pBFR; (3) protocols evaluating potential side effects of pBFR; (4) assessing the applicability and effectiveness of pBFR against/combined with alternative treatment options (e.g., NMES) and (5) evaluating synergistic effects with other early rehabilitation strategies or prolonged pBFR therapy in a home-based rehabilitation setting.

However, the present study failed to report beneficial outcomes in functional rehabilitation and QoL by pBFR. With regard to the published literature, it seems that preoperative physical status has more impact on a successful functional recovery and could be the most important driver for patient satisfaction after surgery [57].

### Limitations

This study presents several potential limitations. The foremost is the relatively small sample size. Nevertheless, the findings from this pilot trial suggest that a pBFR intervention is both feasible and safe following primary and revision TKA surgery, with indications of a beneficial effect on muscle preservation. Regarding the study population, only patients undergoing revision TKA due to aseptic loosening were included. This cohort was selected because these groups represent a large and clinically relevant subset of the TKA population, exhibiting comparable morbidity and mortality profiles comparable to those of primary TKA patients and the general population [58]. Importantly, the postoperative care and rehabilitation protocols following revision TKA for mechanical loosening are equivalent to those applied after primary TKA. However, in cases of PJI, patients experience markedly greater morbidity, mortality, and prolonged hospitalization [59]. Therefore, the present findings may not be directly generalizable to all patient groups; however, evaluating the effects of pBFR in revision procedures due to PJI represents a particularly relevant topic for future research. Another limitation to consider is that rehabilitation following TKA is a multifactorial process influenced by surgical, patient-specific, and rehabilitation-related factors. To minimize the surgical impact on clinical outcomes, we included only two implant types, thereby reducing implant-specific variability. However, the influence of other implant designs and biomechanical considerations (e.g., kinematic alignment and the medial “ball-in-socket” principle with lateral rollback) may significantly affect long-term functional recovery. A third limitation is the lack of data on patient activity levels and intensity during the postoperative period and throughout rehabilitation. Future studies should aim to monitor postoperative patient activity to provide more comprehensive insights into the effects of prehabilitation on postoperative daily activity. Lastly, the study was limited to a follow-up period of three months. While early effects on muscle preservation and joint swelling were observed, the long-term implications for functional recovery and patient satisfaction remain unknown. Therefore, future studies with extended follow-up periods are required to assess the durability of these findings.

## Figures and Tables

**Table 1:** The Knee Injury and Osteoarthritis Outcome Score (KOOS) with five sub-parameters (activities of daily living (ADL), pain, quality of life (QOL), functionality in sports and recovery (SportRec), and Symptoms) during pre- and postoperative period.

Item	Group	T0	T2	T3	*p* Values from Mixed Effect Model		
					Time	Group	Time x Group
ADL	CON	42.5 ± 18.0	57.3 ± 14.2	66.6 ± 15.1	<0.001 (T0 < T2 < T3)	0.495	0.641
	INT	49.4 ± 15.6	60.0 ± 11.6	67.5 ± 13.5			
Pain	CON	41.8 ± 18.7	52.0 ± 13.3	59.8 ± 15.2	<0.001 (T0 < T2 ≈ T3)	0.300	0.963
	INT	48.6 ± 16.4	57.0 ± 12.9	65.0 ± 17.9			
QOL	CON	18.8 ± 14.1	30.1 ± 15.3	40.0 ± 17.0	<0.001 (T0 < T2 < T3)	0.234	0.984
	INT	25.0 ± 16.4	35.1 ± 11.2	45.9 ± 10.5			
SportRec	CON	20.5 ± 23.8	20.6 ± 17.4	33.3 ± 20.2	<0.001 (T0 ≈ T2 < T3)	0.600	0.810
	INT	22.0 ± 25.2	24.0 ± 18.1	40.3 ± 20.4			
Symptoms	CON	39.1 ± 17.1	50.6 ± 13.9	58.9 ± 20.4	<0.001 (T0 < T2 < T3)	0.523	0.721
	INT	42.3 ± 11.8	51.4 ± 17.5	65.9 ± 19.4			

Data are provided as mean ± standard deviation. T0, before surgery, T2, six weeks post-surgery, T3, three months post-surgery. Significant differences between time points are indicated as follows: ≈ (*p* > 0.05) and < (*p* < 0.05).

**Table 2:** Subscores of short-form 36 health survey questionnaire (SF-36) during pre- and postoperative period. The questionnaire consists of eight scales (physical functioning (PF), role limitations due to physical problems (RP), bodily pain (BP), general health perceptions (GH), Vitality (VT), social functioning (SF), role limitations due to emotional problems (RE), and mental health (MH)) that are summarized in two dimensions (physical component summary (PCS) and mental component summary (MCS)).

Item	Group	T0	T2	T3	*p* Values from Mixed Effect Model		
					Time	Group	Time x Group
PF	CON	33.2 ± 30.0	36.0 ± 24.1	57.9 ± 22.5	<0.001 (T0 ≈ T2 < T3)	0.766	0.262
	INT	31.3 ± 16.4	47.7 ± 17.7	54.6 ± 15.4			
RP	CON	33.3 ± 40.8	35.8 ± 31.4	65.0 ± 33.8	0.006 (T0 ≈ T2 < T3)	0.657	0.701
	INT	42.5 ± 42.6	45.8 ± 36.5	62.5 ± 33.9			
BP	CON	20.1 ± 17.1	37.7 ± 14.2	50.8 ± 21.4	<0.001 (T0 < T2 ≈ T3)	0.025 (CON < INT)	0.309
	INT	40.6 ± 16.2	54.5 ± 20.0	58.8 ± 21.2			
GH	CON	54.5 ±11.6	59.3 ± 10.3	57.4 ± 13.2	0.298	0.762	0.767
	INT	52.1 ±22.1	55.2 ± 23.7	57.7 ± 20.5			
VT	CON	47.0 ± 12.3	49.7 ± 12.4	59.0 ± 17.3	0.002 (T0 < T3)	0.786	0.843
	INT	47.5 ± 26.5	53.7 ± 19.6	60.0 ± 15.8			
SF	CON	50.0 ± 27.0	61.3 ± 23.9	72.5 ± 29.3	0.014 (T0 < T3)	0.524	0.923
	INT	58.8 ± 31.8	66.3 ± 28.9	76.3 ± 23.9			
RE	CON	53.3 ± 50.2	76.7 ± 38.7	76.7 ± 38.7	0.005 (T0 < T2 ≈ T3)	0.821	0.805
	INT	46.7 ± 47.7	70.0 ± 39.9	80.0 ± 32.2			
MH	CON	62.0 ± 23.3	66.5 ± 21.0	69.6 ± 21.2	0.104	0.735	0.984
	INT	65.5 ± 24.6	68.8 ± 21.7	72.7 ± 22.1			
PCS	CON	28.2 ±11.2	34.5 ± 7.6	41.5 ± 10.6	<0.001 (T0 < T2 ≈ T3)	0.167	0.524
	INT	32.6 ± 8.98	41.0 ± 8.4	42.0 ± 10.5			
MCS	CON	44.4 ± 16.9	54.2 ± 12.5	54.9 ± 11.4	<0.001 (T0 < T2 ≈ T3)	0.906	0.566
	INT	45.3 ± 16.7	52.1 ± 13.7	58.1 ± 12.4			
RV	CON	3.30 ± 1.06	2.50 ± 1.08	3.00 ± 0.67	0.593	0.963	0.052
	INT	2.67 ± 0.71	3.10 ± 0.99	3.10 ± 0.74			

Data are provided as mean ± standard deviation. T0, before surgery, T2, six weeks post-surgery, T3, three months post-surgery. Significant differences between time points or between groups are indicated as follows: ≈ (*p* > 0.05) and < (*p* < 0.05).

## Data Availability

The data that support the findings of this study are available from the corresponding author upon reasonable request. Source data underlying all Figures and Tables are provided as a Source.

## Abbreviations

The following abbreviations are used in this manuscript:

ACL	Anterior Cruciate Ligament
BFR	Blood Flow Restriction
CON	Control Group
CPM	Continuous Passive Motion
CRT	Chair Rising Test
DXA	Dual-Energy X-Ray Absorptiometry
INT	Intervention Group
KOOS	Knee Injury and Osteoarthritis Outcome Score
LOP	Limb Occlusion Pressure
MCIDs	Minimal clinically important differences
MURF-1	Muscle RING-finger protein-1
NMES	Neuromuscular electrical stimulation
pBFR	Passive Blood Flow Restriction
PJI	Periprosthetic Joint Infection
QoL	Quality of Life
rTKA	Revision Total Knee Arthroplasty
SF-36	Short-Form 36 Score
TKA	Total Knee Arthroplasty
VAS	Visual Analog Scale
6 MWT	Six-Minute Walking Test
